# Recognition of spider mite infestations in jujube trees based on spectral-spatial clustering of hyperspectral images from UAVs

**DOI:** 10.3389/fpls.2023.1078676

**Published:** 2023-02-02

**Authors:** Yue Wu, Xican Li, Qing Zhang, Xiaozhen Zhou, Hongbin Qiu, Panpan Wang

**Affiliations:** ^1^ College of Information Science and Engineering, Shandong Agricultural University, Taian, China; ^2^ Key Laboratory of Digital Earth Science, Aerospace Information Research Institute, Chinese Academy of Sciences, Beijing, China; ^3^ Institute of Agricultural Sciences, the 14th Division of Xinjiang Production and Construction Corps, Kunyu, China

**Keywords:** hyperspectral images, spider mite recognition, spectral features, spatial information, clustering

## Abstract

Spider mite infestations are a serious hazard for jujube trees in China. The use of remote sensing technology to evaluate the health of jujube trees in large-scale intensive agricultural production is an effective means of agricultural control. Hyperspectral remote sensing has a higher spectral resolution and richer spectral information than conventional multispectral remote sensing, which improves the detection of crop pests and diseases. We used hyperspectral remote sensing data from jujube fields infested with spider mite in Hotan Prefecture, Xinjiang to evaluate their use in monitoring this important pest. We fused spectral and spatial information from the hyperspectral images and propose a method of recognizing spider mite infestations of jujube trees. Our method is based on the construction of spectral features, the fusion of spatial information and clustering of these spectral–spatial features. We evaluated the effect of different spectral–spatial features and different clustering methods on the recognition of spider mite in jujube trees. The experimental results showed that the overall accuracy of the method for the recognition of spider mites was >93% and the overall accuracy of the band clustering–density peak clustering model for the recognition of spider mite reached 96.13%. This method can be applied to the control of jujube spider mites in agricultural production.

## 1 Introduction

Jujube spider mites are one of the most common pests affecting jujube trees. Jujube spider mite infestations are explosive, fast-spreading and difficult to control. The leaves of jujube trees infected with spider mites show symptoms such as yellowing, disease spots and curling, which damage normal growth and fruiting and seriously restrict the development of the jujube industry. Conventional control methods use large-scale spraying of pesticides to protect jujube trees from spider mites; however, the heavy use of pesticides not only increases the cost of agricultural production, but also poses potential risks to the environment and ecosystems Huang et al. (2018). It is therefore important to obtain accurate information about the pests and diseases of jujube trees to achieve rapid and accurate agricultural control that balances ecological protection and agricultural production (Singh et al., 2020). Pest monitoring and forecasting for jujube trees is still mainly based on traditional field surveys by plant protection personnel (Tang et al., 2015), which is reliable, but time-consuming, laborious, subjective, has poor timeliness and is unable to obtain global pest information simultaneously. It is therefore difficult to meet the current needs for pest control in large-scale planting areas and the realization of precision agriculture (Zhang et al., 2012).

Remote sensing technology, which is time-sensitive, nondestructive and provides simultaneous observations over wide areas, is commonly used in agriculture (Zhang et al., 2021). Remote sensing can rapidly and accurately recognize and monitor crop pests and diseases, providing an effective means for the timely control of crop pests and diseases (Yuan et al., 2019). The use of remote sensing technology to monitor pests and diseases is based on airborne cameras and radiometers, which record the reflectance characteristics of observed features to recognize pest and disease infestations (Jiang et al., 2021). Remote sensing technology recognizes and distinguishes various types of targets using spectral characteristics. Hyperspectral data have a wide band range and high spectral resolution, which can simultaneously obtain high-resolution spectral information and spatial information for the more effective recognition and diagnosis of crop pests and diseases (Lan et al., 2019). In general, when crops are stressed by pests and diseases, the tissue structure, water content and chlorophyll of the crop canopy are damaged to varying degrees and the spectral characteristics of the crop change accordingly (Xu et al., 2022). Crop pests and diseases can be recognized by the differences in spectral reflectance between healthy and infected crops.

Near-Earth remote sensing avoids interference from atmospheric and terrain factors and can be used to obtain accurate spectral characteristics of crops and to calibrate the target spectra acquired by satellites and unmanned aerial vehicles (UAVs). Chen et al. (2007) analyzed the canopy spectrum of cotton yellow wilt and found that the visible band (680–760 nm) and the near-infrared band (731–1371 nm) showed a significant response to yellow wilt infestation. They used these two response regions to construct a prediction model for cotton canopy yellow wilt with a prediction accuracy of 82%. Furlanetto et al. (2021) used principal components analysis (PCA) to extract spectral features from 87 bands in soybean leaves and combined different bands to diagnose soybean rust by linear discriminant analysis.

Ground-based remote sensing is limited to collecting the spectral reflectance of a single point and it is therefore difficult to monitor crop pests and diseases in a large area at the same time. By contrast, satellite remote sensing can obtain remote sensing information with multiple time–space–spectrum resolutions simultaneously to rapidly and accurately monitor the development of pests and diseases. Prabhakar et al. used Sentinel-2 satellite data to quantitatively invert and model the relationships between the leaf area index, the above-ground biomass, the yield and the spectral vegetation index between healthy and pest-infested farms using ANOVA with coefficients of determination >0.80 (Prabhakar et al., 2022). Li et al. (2020) selected five vegetation indices to accurately recognize root rot of cotton in Sentinel-2 images using binary logistic regression models with a best recognition accuracy of 92.95%. Satellite remote sensing can rapidly monitor the occurrence of pests and diseases over time in large areas, but is restricted by image resolution, clouds and rain.

The UAV pest monitoring platform has the advantage of convenient data acquisition. UAVs can carry a hyperspectrometer to obtain remote sensing data with a high spatial and spectral resolution, which is an effective supplement to satellite remote sensing. Huang et al. (2007) used a regression model to assess the accuracy of photochemical indices to quantify the disease index of wheat stripe rust and established a linear model of photochemical indices and a disease index of stripe rust in aerial remote sensing images in different time periods to detect wheat stripe rust in aerial hyperspectral images. However, the imaging principle of hyperspectral images is complex and relying only on spectral information to recognize crop pests and diseases generates a lot of pretzel noise. The recognition accuracy can be improved by incorporating spatial information. Guo et al. (2021) extracted vegetation indices and texture features from hyperspectral images of UAVs and developed monitoring models for yellow rot using partial least-squares regression. These studies combined the texture information in the images to compensate for the shortcomings of using spectral information alone, but the adopted spectral features were based on the vegetation indices of fixed-band combinations and it is difficult to take advantage of the rich, multi-band hyperspectral information.

Liu et al. (2020) extracted the original spectral bands, vegetation indices and texture features from hyperspectral images and monitored wheat blast using a back-propagation neural network, which constructed the spectral features using interval sampling to select the feature bands with greater chance and randomness. Compared with RGB and multispectral images, the continuous rich spectral information of hyperspectral images more realistically reflects the subtle differences in the reflection characteristics of surface materials. Hyperspectral images have strong inter-band correlation and contain a large amount of redundant information. This study investigated how to scientifically extract spectral features and incorporate spatial information to build a highly accurate pest monitoring recognition model.

We used hyperspectral images acquired by a low-altitude UAV with a high spectral and spatial resolution, which avoided the problem of mixed pixels (Ma et al., 2019). In processing hyperspectral images, the workload of tagging jujube tree sample information in images is large because, as the data dimension (i.e., the number of image bands) increases, the amount of annotation data required has to increase exponentially (Pothen and Pai, 2020; Wang et al., 2022). To solve the problem of too few samples and too high a number of hyperspectral image bands in supervised learning, we used the unsupervised classification method to cluster each image element into different clusters according to its attributes and distance in the feature space without a large amount of sample data (Zhang et al., 2018).

We explored the application of UAV hyperspectral remote sensing in jujube pest recognition and propose a method of recognizing jujube pests by fusing spectral features and spatial information. The pixels in remote sensing images were used as the basic classification unit to mine the spectral features and spatial information of the jujube canopy, which improved the recognition accuracy of jujube spider mite. We first compared the effects of different spectral feature construction methods on the accuracy of jujube pest recognition using hyperspectral data. We then evaluated the effects of different unsupervised learning methods—K-means, fuzzy C-mean (FCM) clustering and density peak clustering (DP)—combined with spectral–spatial features on the accuracy of jujube pest recognition. We propose a hyperspectral image recognition method for jujube pests based on constructing spectral features, fusing spatial information and clustering algorithms.

The contributions of this article can be summarized from three aspects. First, conventional monitoring methods are inadequate in terms of hyperspectral information utilization, this study fully exploited the spectral information using different dimensionality reduction methods and utilized the advantages of hyperspectral resolution for UAV monitoring of pests and diseases. Second, the spectral-spatial information of hyperspectral images was extracted by weighted spatial-spectral mean filtering algorithm, we enhance the spatial correlation of image recognition and apply it in the monitoring of pests and diseases. Third, we compare different feature construction and clustering algorithms, this study constructs an effective, rapid and highly accurate model for recognition of jujube spider mite infestations.

## 2 Materials and methods

### 2.1 Study site

**Figure 1:**
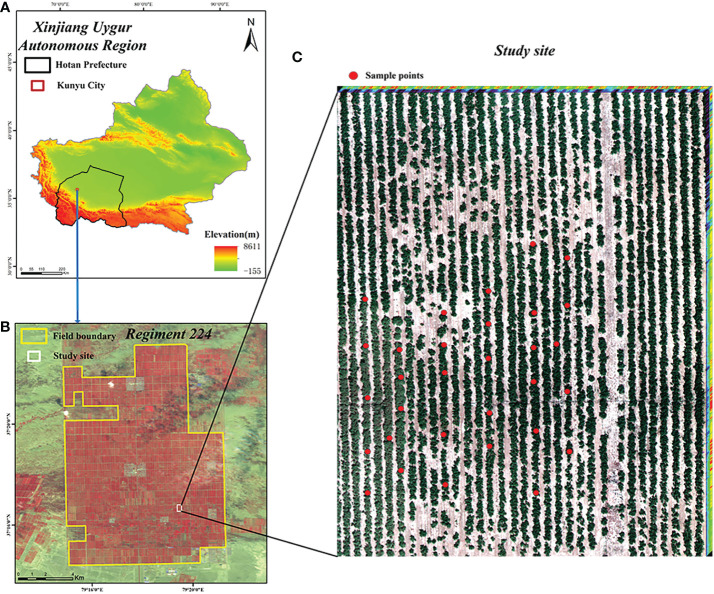
Location of the study site. **(A)** Xinjiang Uygur Autonomous; **(B)** Regiment 224; **(C)** Study site.

The study area is located in the 224th Regiment of Xinjiang Production and Construction Corps, Hotan Prefecture, Xinjiang (**Figure 1**), at the northern foot of the Karakorum Mountains and the southern edge of the Tarim Basin, with high terrain in the south and low terrain in the north. It has a warm temperate continental desert climate, with year-round drought and little rain, sufficient light for crop growth, total annual sunshine of 2769.5 h, abundant heat, a year-round average temperature of 12°C and large differences in temperature between day and night. The region is suitable for the growth of jujube trees and the accumulation of jujube sugar. The study area is approximately 234.75 km^2^ in size and jujube trees are the main crop, with a cultivation area of about 90 km^2^. The jujube trees in the field are often threatened by outbreaks and the spread of spider mites and other pests and diseases due to the wide and dense distribution of jujube trees in the single-species cultivation area.

### 2.2 Data acquisition and pre-processing

Data were collected from July 28 to August 6, 2021, during the critical growth period of jujube trees when they are susceptible to pests and diseases. We investigated the infestations status of jujube fields and selected abandoned jujube fields with serious spider mite infestations and normal jujube fields with less infestations as the experimental area. We planned a reasonable UVA flight route, data acquisition period ranged from 11:00 to 15:00 (the sun altitude angle was >45°) under sufficient daylight or cloudless conditions.

A DJI M600pro UAV equipped with a Rikola hyperspectrometer was used to collect hyperspectral image data from jujube date palm trees in a typical area. The image pixel array of the Rikola hyperspectrometer is 1010 × 1010 and this UAV remote sensing platform is sufficiently convenient and flexible to adapt to complex flight environments. The hyperspectral cameras were radiometrically corrected with diffuse reflectance gray plate, four 50cm×50cm diffuse reflectance gray plates (reflectance of 3%, 22%, 48%, 64%) were placed on the horizontal surface of the test location while ensuring that there were no shadows and interfering objects on the surface of the plate. The UAV course overlap and side-phase overlap were 75%, the flight speed was 6 m/s, the flight altitude was 80 m, the spectral resolution was 10 nm, the wavelength range was 500–950 nm, the number of channels was 45 and the spatial resolution was up to 6 cm (see **Table 1**).

**Table 1:** Primary parameters of unmanned aerial vehicle hyperspectral imaging system.

Parameters	Specification
**Weight**	720g
**FOV**	36.5°
**Spatial resolution**	6cm
**Spectral resolution**	10nm
**Spectral range**	450-900nm
**Bands**	45
**FWHM**	5-13nm
**Physical pixel size**	5.5um
**Flight altitude**	80m
**Flight velocity**	12m/s
**Image resolution**	1010×1010

The spectral reflectance and health status of 30 sets of samples, consisting of a total of 90 samples, were gathered and recorded in the study area as a validation of the recognition results. As a control, the ratio of infected samples to healthy samples was around 1:2 (the gathered data contained 30 infected samples and 60 healthy samples). The spectrum of each sample was repeated five times, the average of the spectral reflectance of the five acquisitions was regarded as the spectral reflectance of the sample, the sampling point was located using a hand-held GPS device and labeled in the image with ENVI. Each sample is a small area containing multiple pixels, and the ROI of healthy and infected is made by combining GPS positioning to determine the image points corresponding to the sample points as a validation set for the recognition results. 5,036 pixels of healthy area, 2,438 pixels of infected area and 11,213 pixels of ground were labeled. The overall accuracy and kappa coefficient was calculated using the confusion matrix to assess the accuracy of the model for recognition of jujube spider mite infestations.

The images were noisy due to the influence of the instruments, environment and measurement methods during image acquisition and the hyperspectral images were first corrected to eliminate the dark currents generated by the sensor unit, then corrected for vignetting and geometric distortion with Nikon Capture NX-D software, stitched with Pix4Dmapper software and the alignment accuracy was improved using ground control points. The reflectance of the soil background can interfere with the spectral reflectance of the jujube canopy as a result of the complex spatial heterogeneity of the soil composition (Yue et al., 2015; Gao et al., 2018). Using spectral indices for extraction of the jujube area and masking the soil background can effectively avoid this interference while improving the operational efficiency of the algorithm. We compared the effects of the visible vegetation difference index and the normalized vegetation index on the extraction of information about the jujube canopy. Both vegetation indices could be effectively extracted and the visible vegetation difference index gave a better elimination of the effect of the shadow of the jujube trees than the normalized vegetation index (Lu et al., 2018).

### 2.3 Methods

#### 2.3.1 Recognition of jujube spider mite

**Figure 2:**
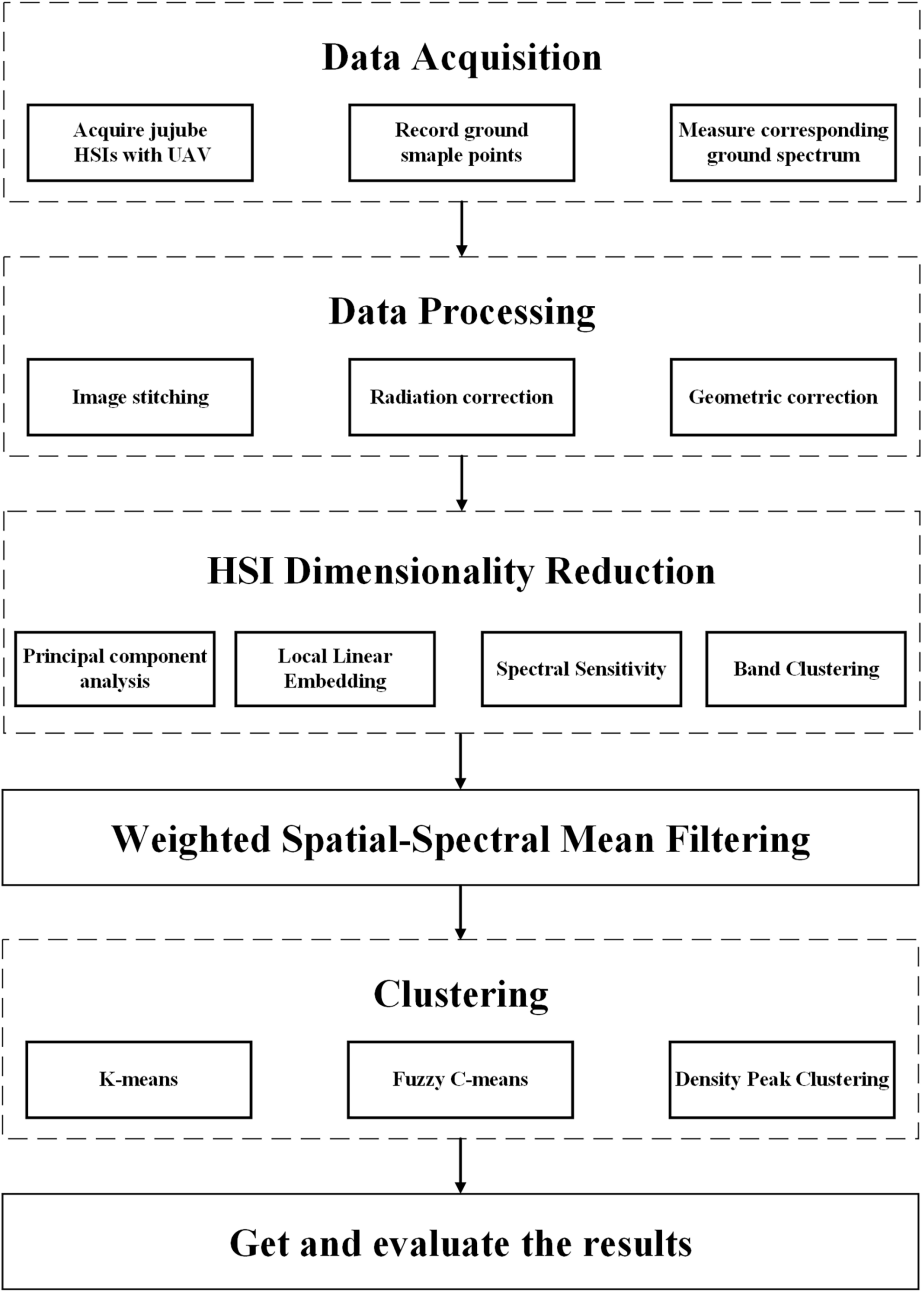
Flow chart of recognition of jujube spider mite infestations.

**Figure 3:**
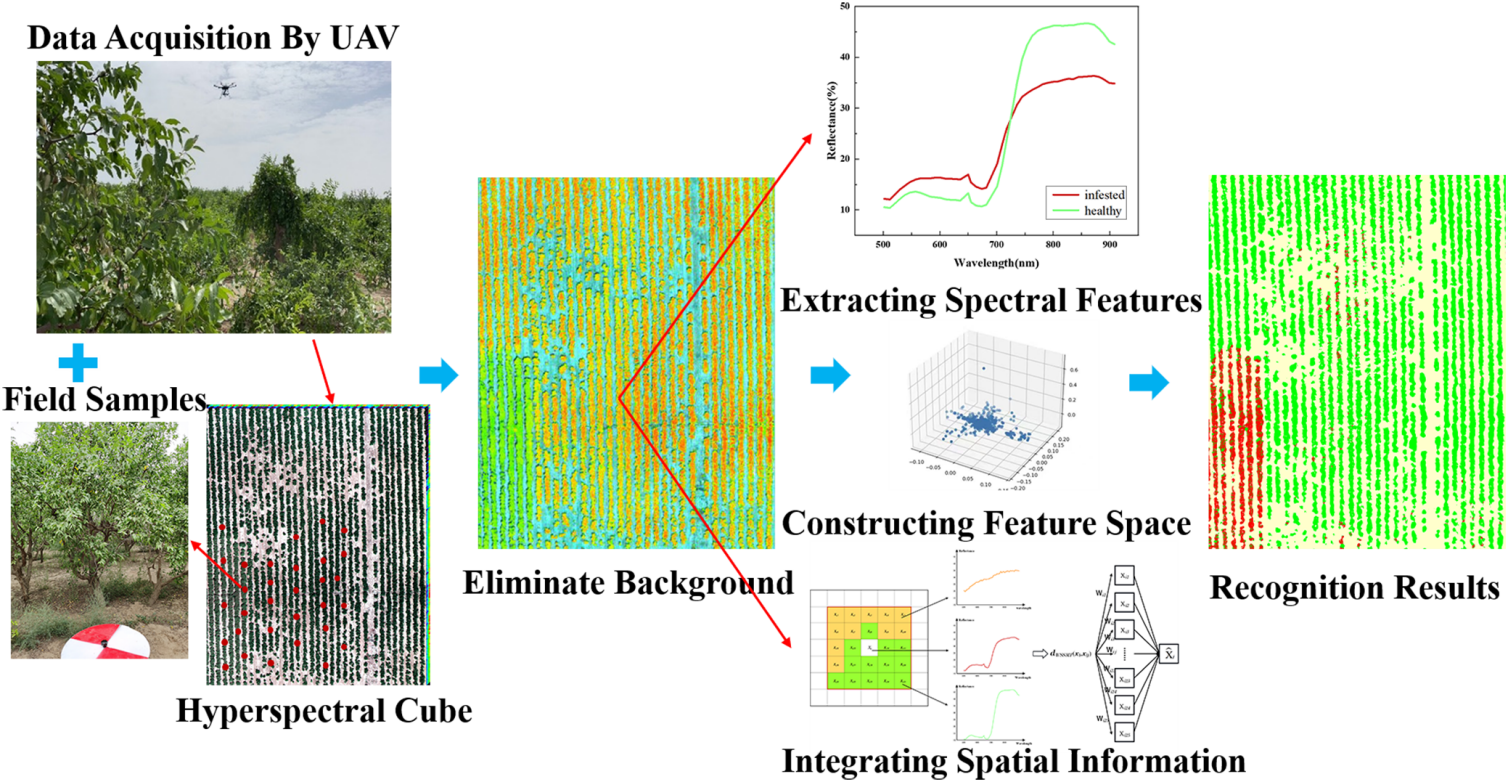
Framework of recognition program of jujube spider mite infestations.

The process of jujube spider mite pest recognition by different spectral feature construction methods combined with different clustering algorithms (**Figures 2**, **3**) consists of six steps: (1) data acquisition (the simultaneous acquisition of jujube hyperspectral images and ground sample points); (2) data pre-processing (e.g., image stitching, geometric correction, radiation correction, background extraction); (3) dimensionality reduction of the jujube hyperspectral images by feature extraction and selection, and the construction of the spectral feature space of jujube trees using four methods—PCA, local linear embedding (LLE), spectral sensitivity and band clustering; (4) fusion of the spatial information from jujube images using a weighted spatial–spectral mean filtering algorithm; (5) clustering of the spectral features of the fused spatial information from the jujube trees using three clustering methods (K-means, FCM and density peak clustering) to obtain identification results; and (6) assessment of recognition results of jujube spider mite infestations based on ground sampling.

The proposed algorithms run on python 3.9 and Windows 10 as the operating system and on a GeForce RTX 3060 GPU with 12 GB of video memory and an Intel(R) Core (TM) i7-12700F CPU, accuracy evaluation was accomplished in ENVI5.3.

#### 2.3.2 Feature construction methods

Hyperspectral data are high dimensional, rich in waveband information and have a higher spectral resolution than multispectral data. However, tens or even hundreds of bands of hyperspectral data bring great challenges to the classification and recognition of ground targets (Huang et al., 2020). Building recognition models directly with raw hyperspectral data greatly increases the computational complexity and high-dimensional data will also attenuate the distance effect, making it difficult to aggregate sample points and affecting the accuracy and performance of the classification model (Li et al., 2017). It is therefore common to use feature extraction and feature selection to remove redundant data and transform hyperspectral data from a high-dimensional space to a low-dimensional subspace (Wang et al., 2018). Information that contributes significantly to feature identification is retained and information with a high linear correlation, redundancy and low contribution to feature identification is removed (Liang et al., 2016). Feature extraction and feature selection to compress high-dimensional data are realized from different perspectives and we therefore evaluated the effects of four different ways of constructing spectral features (i.e., PCA, LLE, spectral sensitivity and band clustering) on the accuracy of jujube spider mite pest recognition.

(1) Feature extraction

The feature extraction method is a linear or nonlinear transformation of the original hyperspectral image. Assuming that ${\left\{x_{i}\right\}}_{i=1}^{N}$ represents the hyperspectral data, where *N* represents the total number of image elements and *x_i_* ε *R^d^* represents the high-dimensional data, then feature extraction is the projection of high-dimensional data into a low-dimensional space that *R^d^* →*R^f^* and obtain the optimum combination of the bands, where *d* and *f* are the number of dimensions of the data—that is, the number of bands in the image. The advantage of the dimensionality reduction method for feature extraction is that it fuses information from all bands to avoid the loss of spectral information, but it also destroys the original structural properties among the hyperspectral bands and the interpretability of the data after dimensionality reduction becomes poor (Pascucci et al., 2020; Yanjun et al., 2020; Tang et al., 2022).

① Linear dimensionality reduction algorithm: principal components analysis

Principal components analysis (PCA) is one of the most commonly used algorithms for the feature extraction of hyperspectral data and is also an effective method for dimensionality reduction to solve the problem of high correlation and multicollinearity among data. PCA greatly reduces the amount of data by decomposing the covariance eigenvalues in the data. PCA projects the original data onto a new coordinate system using linear projection to remove redundant spectral information and retain the main characteristics in the data (Kang et al., 2021a).

② Nonlinear dimensionality reduction algorithm: local linear embedding

Local linear embedding is an important nonlinear manifold learning method. Linear dimensionality reduction techniques such as PCA have a good effect on dimensionality reduction for data with relatively low dimensionality and a global linear structure, whereas the processing of nonlinear data can make the data lose their original structure and is unable to reflect the nonlinear characteristics. Hyperspectral data inherently exhibit nonlinear data characteristics as a result of ground scattering, the representation model as a bidirectional reflectance distribution function, and the heterogeneity of multiple scattering and subpixel components within the pixel. Compared with linear dimensionality reduction algorithms such as PCA, LLE algorithms have the advantage of applying nonlinear feature analysis of high-dimensional data to find simple, low-dimensional expressions of manifold structures in complex, high-dimensional nonlinear structures (Yue et al., 2019).

(2) Feature selection

The band selection process selects a subset of the spectral feature space in the hyperspectral image based on different criteria and characterizes the hyperspectral data in all bands with a representative combination of bands. This can maximize the distinction between other targets while removing a large amount of redundant information from the hyperspectral data. Band selection is the selection and rejection of spectral bands, which can better preserve their original spectral reflectance properties and physical meaning, and is generally achieved based on the separability between bands or the amount of information between bands (Jing et al., 2010). In agricultural remote sensing, linear regression is normally used to select the band with a strong correlation with the measured data by using the reflectance and waveform characteristics of the spectral curve. We used spectral sensitivity and band clustering to select the characteristic bands that can effectively distinguish jujube spider mite infestations.

① Spectral sensitivity

The spectral sensitivity reflects the difference in reflectance between healthy and stressed plants at different wavelengths and is calculated as the ratio of the spectral difference between the stressed and normal plants to the normal plants. The larger the absolute value of the spectral sensitivity, the simpler it is to distinguish infected crops from each other in this band. Kobayashi et al. (2001) used spectral sensitivity to extract regions of significant differences in the spectral response of rice plants subjected to stress. In hyperspectral data, the number of bands is large and the correlation between bands with similar wavelengths is strong. The equation (1) is used to calculate the absolute value of the spectral sensitivity of each band and the band with the greatest spectral sensitivity and largest distance in wavelength is selected as the characteristic band for identifying the infected crops:


(1) $$S=\frac{Ss-Sh}{Sh}$$

where *S* is the spectral sensitivity, *Ss* is the spectral reflectance of the stressed plants and *Sh* is the spectral reflectance of the normal plants.

② Band selection based on clustering

Band selection of hyperspectral data can be divided into supervised, semi-supervised and unsupervised methods according to the availability of the corresponding samples for training. However, in practice, it is hard to obtain sufficient ground sample information. We therefore used the unsupervised band selection method, which considers the amount of information and the correlation of the image data bands themselves and clusters them in the whole spectral interval to form a band subspace. The band selection methods commonly used for hyperspectral data include the optimum index factor method, the adaptive band selection method and the automatic subspace partition (ASP) method.

Traditional band selection used the standard deviation and correlation coefficient to construct the optimum index factor to select the optimum band combination based on the information content of each band, but the computational effort required to calculate the correlation coefficient among the bands of hyperspectral data is too large for this method to be used for hyperspectral band selection. Then adaptive band selection method was proposed, which calculates the ratio of the standard deviation of each band to the correlation coefficient of the adjacent bands as an index to select the best band, which reduces the computational effort (Baisantry et al., 2021; Kang et al., 2021b). However, the adaptive band selection method does not consider the connection between the candidate bands and the non-adjacent bands and therefore the selected band combination may not be the optimum solution (Yang and Kan, 2019). We therefore used an ASP method for the band selection of hyperspectral images of jujube trees. The ASP algorithm divides the hyperspectral data space into multiple subspaces by defining the inter-band correlation coefficient matrix and the nearest neighbor transferable correlation vector according to the band space relationship of the spectrum. The algorithm then selects the most representative bands from the spectral space (Zhang and Liu, 2020).

#### 2.3.3 Weighted spatial–spectral mean filtering

In the process of hyperspectral data acquisition, which is influenced by both the environment and sensors, the acquired spectral reflectance inevitably contains errors. Most of the current recognition and classification methods only utilize the spectral information in the hyperspectral data and ignore the correlation of pixels in space. This is prone to pepper noise and the misclassification of objects and largely affects the accuracy of recognition and classification (Du et al., 2016). The full utilization of spatial information in hyperspectral images can partially alleviate the effects of errors. We therefore used the WSSMF algorithm to improve the accuracy of the jujube pest recognition algorithm (Zhao et al., 2012).

**Figure 4:**
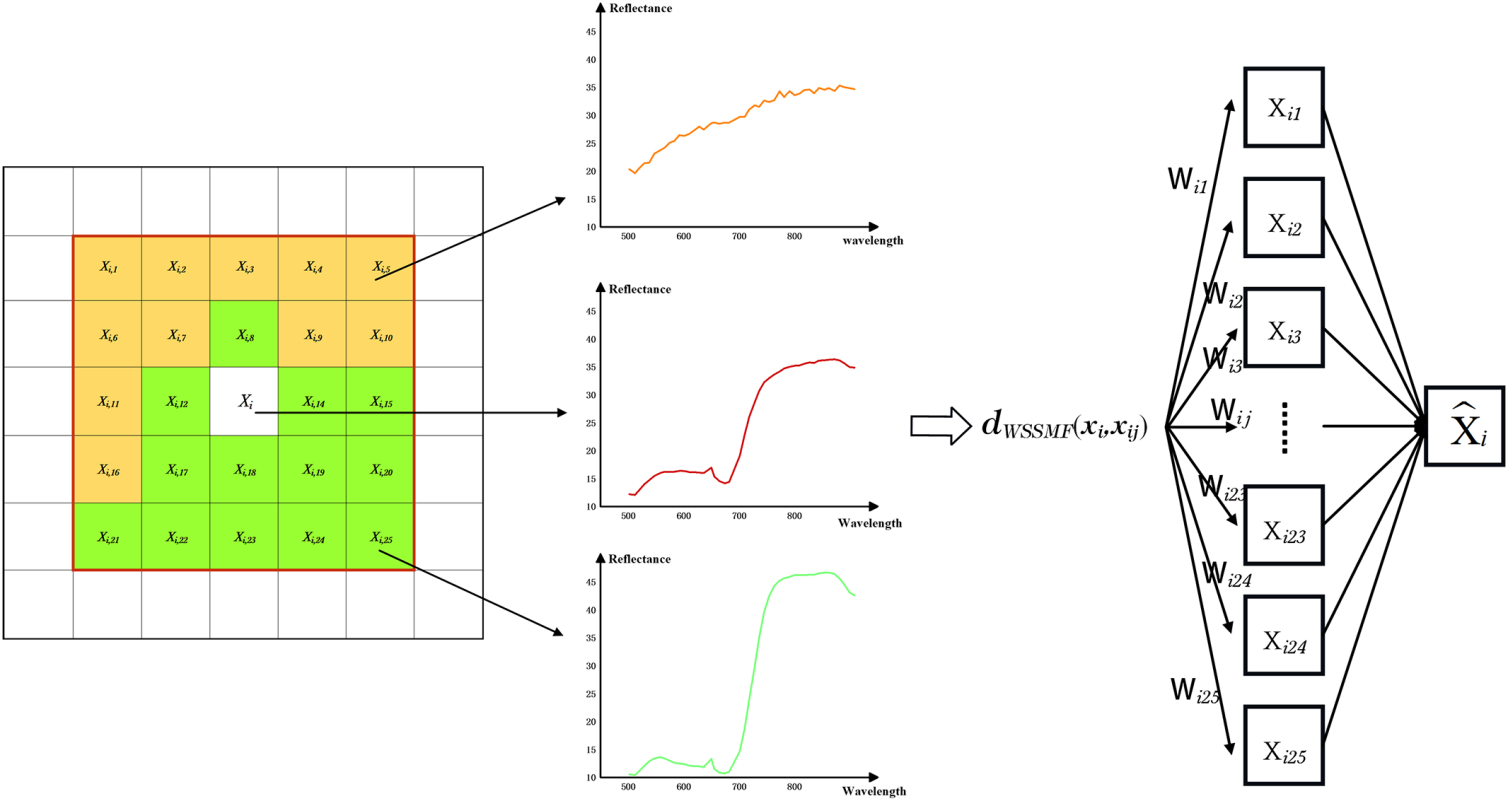
Weighted Spatial-Spectral Mean Filtering algorithm.


We generally consider adjacent pixels in the same area of an image to be of the same object type. We used the weighted spatial–spectral mean filtering algorithm and the WSSMF algorithm reconstructed the central pixel point by measuring the spectral similarity between the central pixel and the pixels in the neighborhood (Kang et al., 2013; Zeng et al., 2018) (**Figure 4**). For a given pixel point *x_i_*, we define the window size *w* with *x_i_* as the center and the neighborhood radius *a*=(*w*-1)/2, then the neighborhood area *Ω*(*x_ij_*) can be expressed as Equation (2):

(2) $$\Omega (x_{ij})=\left\{x_{pg}|p\in [i-a,i+a],q\in [i-a,i+a]\right\}$$

If we define *k*=*w* ^2^-1, then the elements in the neighborhood *Ω*(*x_ij_*) are represented as *x_i,_ x_i_*_1_, *x_i_*_2,_ *x_i_*_3_…, *x_ik_*, The size of the weighted mean filter window *w* can be adjusted according to the strength of the spatial distribution features of the image and the weighted spatial–spectral mean filtering of the central pixel point x in combination with the neighboring pixels as Equation (3):


(3) $$x_{ij}=\frac{\sum_{x_{pq}\in \Omega \left(x_{ij}\right)}w_{pq}x_{pq}}{\sum_{x_{pq}\in \Omega \left(x_{ij}\right)}w_{pq}}=\frac{\sum_{p=i-1}^{i+1}\sum_{q=i-1}^{j+1}w_{pq}x_{pq}}{\sum_{p=i-1}^{i+1}\sum_{q=i-1}^{j+1}w_{pq}}$$

where *w_pq_* =*exp*{-γ_0_||*x_ij_*-*x_pq_* ||^2^}+is the weight occupied by neighboring pixels in the neighborhood of the pixel point space and the spectral value of the center pixel is used as the spectral value of all pixels in the neighborhood after weighting according to the distance distribution of the neighboring pixels from the center pixel point *x_i_*, γ_0_ is the spectral factor that adjusts the weight of pixel points between neighbors, which we replaced using the average of the Euclidean distance between the neighboring pixels and the central pixel *x_i_*, i.e., let as Equation (4).


(4) $$\gamma_{0}=\frac{1}{w^{2}}\sum_{k=1}^{w^{2}}\left|\left|x_{ij}-x_{ik}\right|\right|$$

To balance the recognition effect and computational efficiency, we take the filtering window value as 5. The WSSMF algorithm can use the spatial correlation in hyperspectral data to effectively reduce the influence of noise the data, strengthen the spatial correlation between adjacent image elements and effectively improve the spatial continuity of the recognition results.

#### 2.3.4 Hyperspectral image clustering

The classification of hyperspectral remote sensing images includes both supervised and unsupervised classification. In the process of supervised classification, training sample data and *a priori* information are both required and, as a result of the high-dimensional characteristics of hyperspectral data, the amount of sample data required for supervised classification grows exponentially. In practice, it is difficult to obtain a large amount of hyperspectral sample data, which seriously restricts the classification accuracy. In unsupervised classification, the data are classified into different clusters based on the data characteristics only through similarity metrics (Krishnan et al., 2022). With the development of artificial intelligence technology, more and more hyperspectral clustering algorithms have been proposed, but many of them are still in theory, it is difficult to guarantee robustness in application. The robustness of K-means, FCM and DP clustering algorithms have been verified by a large number of applications and experiments. We therefore used clustering algorithms to cluster the spectral features of jujube trees and evaluated the effectiveness of the K-means, FCM and density peak clustering algorithms to recognize the jujube trees infected with spider mite through the extracted spectral–spatial features.

(1) K-means clustering algorithm

K-means is a well-established unsupervised classification algorithm for remote sensing images. It is based on distance metrics and has a low complexity, efficient clustering, excellent scalability and high efficiency in dealing with large amounts of data (Ji et al., 2019).

We used the K-means clustering algorithm to cluster the date palm trees based on their spectral features. The spectral features were divided into k clusters by minimizing the sum of the squared distances between discrete points and the nearest center of mass. In the recognition of jujube spider mites, the cluster centers were set to three—that is, healthy date palm trees, date trees infected with jujube spider mite and the ground surface—and the steps of the K-means clustering algorithm for jujube pest recognition were as follows:

Step 1: Randomly select three points (*c*_1_, *c*_2_ and *c*_3_) from the spectral feature set as the initial center of mass.

Step 2: Calculate the Euclidean distance *d_ij_* =||*k*-*k_c_* ||^2^ between each sample point *X* to each center of mass of the points *c*_1_, *c*_2_ and *c*_3_ and assign the sample points to their nearest clusters.

Step 3: Recalculate the centers of mass of all clusters.

Step 4: Repeat steps 2 and 3 until the cluster point was in a stable position.

(2) Fuzzy C-mean clustering algorithm

The FCM is a division-based clustering algorithm that gives the maximum similarity between objects classified into uniform clusters and the minimum similarity between different clusters. The FCM algorithm characterizes the fuzzy segmentation of images with fuzzy affiliation, defines the non-similarity measure of pixels and clusters as the Euclidean distance, and constructs the objective function from it. It obtains the optimal fuzzy segmentation by solving this objective function, which has the advantage of fast convergence and is widely used in image processing (Yifan et al., 2021). The steps of FCM clustering algorithm for jujube hyperspectral image pest recognition are as follows:

Step 1: Given the number of categories m and the allowable error E, the number of iterations *t*=1.

Step 2: Initialize the distance centers *c*_1_, *c*_2_ and *c*_3_.

Step 3: Calculate the affiliation degree *u*(*t*), $u_{ij}=\frac{{\left[\frac{1}{\left|\left|x_{j}-w_{i}\right|\right|}\right]}^{\frac{1}{m-1}}}{\sum_{k=1}^{c}{\left[\frac{1}{\left|\left|x_{j}-w_{i}\right|\right|}\right]}^{\frac{1}{m-1}}}$ .

Step 4: Correct the clustering center *w*(*t*+1), $w_{i}=\frac{\sum_{j=1}^{n}{\left(u_{\mathrm{ij}}\right)}^{m}x_{j}}{\sum_{j=1}^{n}{\left(u_{\mathrm{ij}}\right)}^{m}},i=1,2,3\ldots \ldots ,c$ .

Step 5: Calculate the error: $e=\sum_{i=1}^{c}||\omega \left(t+1\right)-\omega \left(t\right)|{|}^{2}$ , if *e*< *E*, then the algorithm ends, otherwise *t*=*t*+1, repeat the steps.

(3) Density peak clustering algorithm

Density peak clustering is a density-based clustering method. The algorithm finds the denser points by calculating the density of the data points and calculating the distance of the denser points. The point with both the larger density and larger distance values is determined as the cluster center. Density peak clustering has a better clustering effect on high-dimensional data and datasets with an uneven density distribution (Ge et al., 2021). It is simple and accessible to implement and the clustering results are less sensitive to different parameters. The steps of the density peak clustering algorithm for jujube hyperspectral image pest recognition are as follows:

Step 1: Calculate the sample point distance matrix *d_ij_* =*dist*(*X_i_*, *X_j_*) using the spectral sample set data, where *X_i_* is each data point.

Step 2: Determine the neighborhood truncation distance *d_c_*.

Step 3: Calculate the local density $\rho_{i}=\sum_{x_{j}\in X}\chi \left(dist\left(X_{i},X_{j}\right)-d_{c}\right)$ and the center shifted distance $\delta_{i}=\{\begin{array}{l} \underset{j:\rho_{i}<\rho_{j}}{min}\left(dist\left(X_{i},X_{j}\right)\right),if\exists j\text{ s.t.}\rho_{i}<\rho_{j}\\ \underset{j}{max}\left(dist\left(X_{i},X_{j}\right)\right),otherwise \end{array}$ otherwise for each point.

Step 4: Select the cluster centers and categorize the non-cluster center data points.

#### 2.3.5 Accuracy evaluation

In this study, we use the confusion matrix, overall accuracy (OA), producer accuracy (P) and user accuracy (U) for accuracy evaluation the confusion matrix was obtained from the recognition results and ROI validation samples to calculate the OA, Kappa coefficient, P, and U for each model.

The confusion matrix is a representation used for accuracy evaluation, and the upper and left sides of the confusion matrix respectively represent the ground truth sampled pixel values and model predicted pixel values for health, infection and ground. The recognition accuracy is evaluated by OA and the Kappa coefficient, OA represents the probability that the recognition result is consistent with the corresponding area on the ground, Kappa coefficient is a consistency test indicator using discrete multivariate techniques, P and U respectively evaluate the recognition accuracy of a single category from the perspective of map producers and users. The formulae are as follows:


(5) $$OA=\frac{\sum_{k=1}^{n}p_{kk}}{p\;}$$

where $\sum_{k=1}^{n}p_{kk}$ represents the number of pixels of all samples correctly identified, *p* represents the total number of samples.


(6) $$Kappa=\frac{P_{o}-P_{e}}{1-P_{e}}$$

where *P_o_* represents the overall accuracy, *P_e_* is the hypothetical random agreement.


(7) $$P=\frac{p_{c}}{p_{t}}$$

where *p_c_* represents the correctly recognized samples of a specific class, and *p_t_* is the total number of pixels in the category.


(8) $$U=\frac{p_{c}}{p_{r}}$$

where *p_r_* represents the total number of pixels classified into this category.

## 3 Results

We evaluated and compared the different methods of recognizing jujube spider mite pests with UAV hyperspectral images using ground sample sites as a reference. We used a confusion matrix, the overall classification accuracy and the kappa coefficient as evaluation metrics for the final results.

### 3.1 Spectral response of jujube spider mite infestations

**Figure 5:**
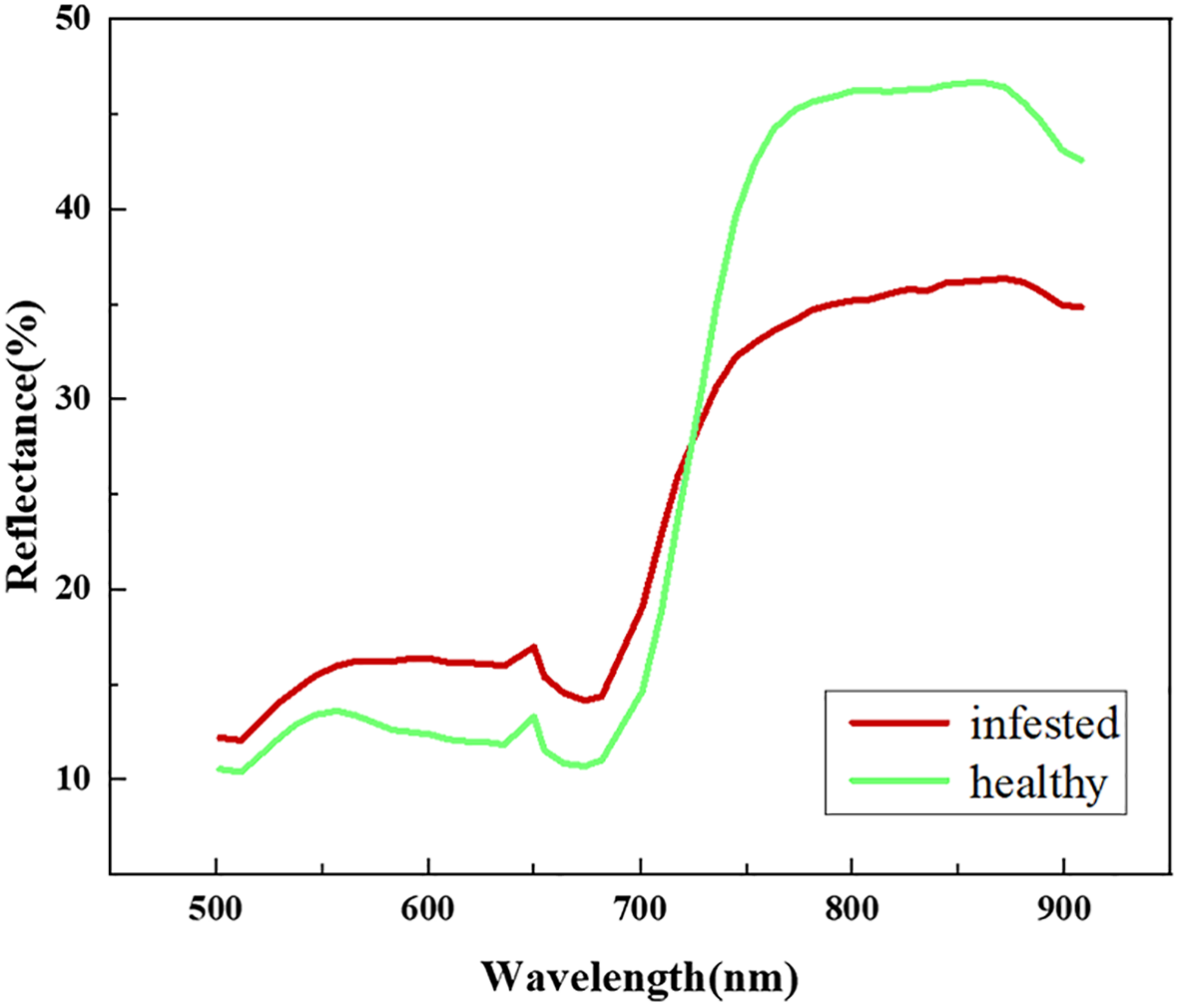
Spectral characteristics of healthy and infected jujube.

**Figure 6:**
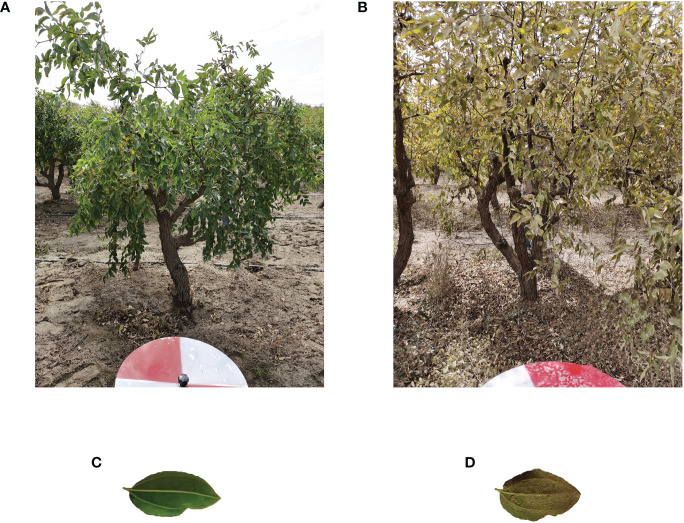
Images of healthy jujube trees and infected jujube trees. **(A)** Healthy jujube tree, **(B)** Infected jujube tree, **(C)** Leaf of healthy jujube trees, **(D)** Leaf of infected jujube trees.

We extracted the spectral reflectance of healthy jujube trees with jujube spider mite infestations recorded on the ground (**Figure 5**). The waveform characteristics of the spectral reflectance of jujube trees infested with spider mite were similar to those of healthy jujube trees, although there were some differences. We collected images of healthy and infected jujube trees to record the differences in the visible, the healthy jujube trees and leaves appeared green while the infected jujube trees and leaves were yellowish (**Figure 6**). The samples infested with jujube spider mite had a higher reflectance in the visible range (450–730 nm) than the healthy jujube samples. By contrast, the reflectance of healthy jujube samples in the near-infrared band (780–900 nm) was much higher than that of the samples infested with jujube spider mite. The differences in the spectral waveform between healthy jujube samples and samples infested with jujube spider mite provide a theoretical basis for the establishment of recognition model of jujube spider mite infestations.

### 3.2 Spectral feature selection and extraction jujube spider mite infestations

We extracted the spectral features sensitive to jujube spider mite pests by feature extraction and feature selection. In the feature extraction process, the principal components of the hyperspectral images were extracted using PCA and their cumulative information contribution was counted. The first four principal components contributed 97% of the information. We extracted the original hyperspectral data with PCA to its first four principal components as spectral features to achieve linear dimensionality reduction of the hyperspectral data. We also extracted four components of the hyperspectral data as spectral features for the next step of classification and recognition using the LLE nonlinear dimensionality reduction algorithm.

**Figure 7:**
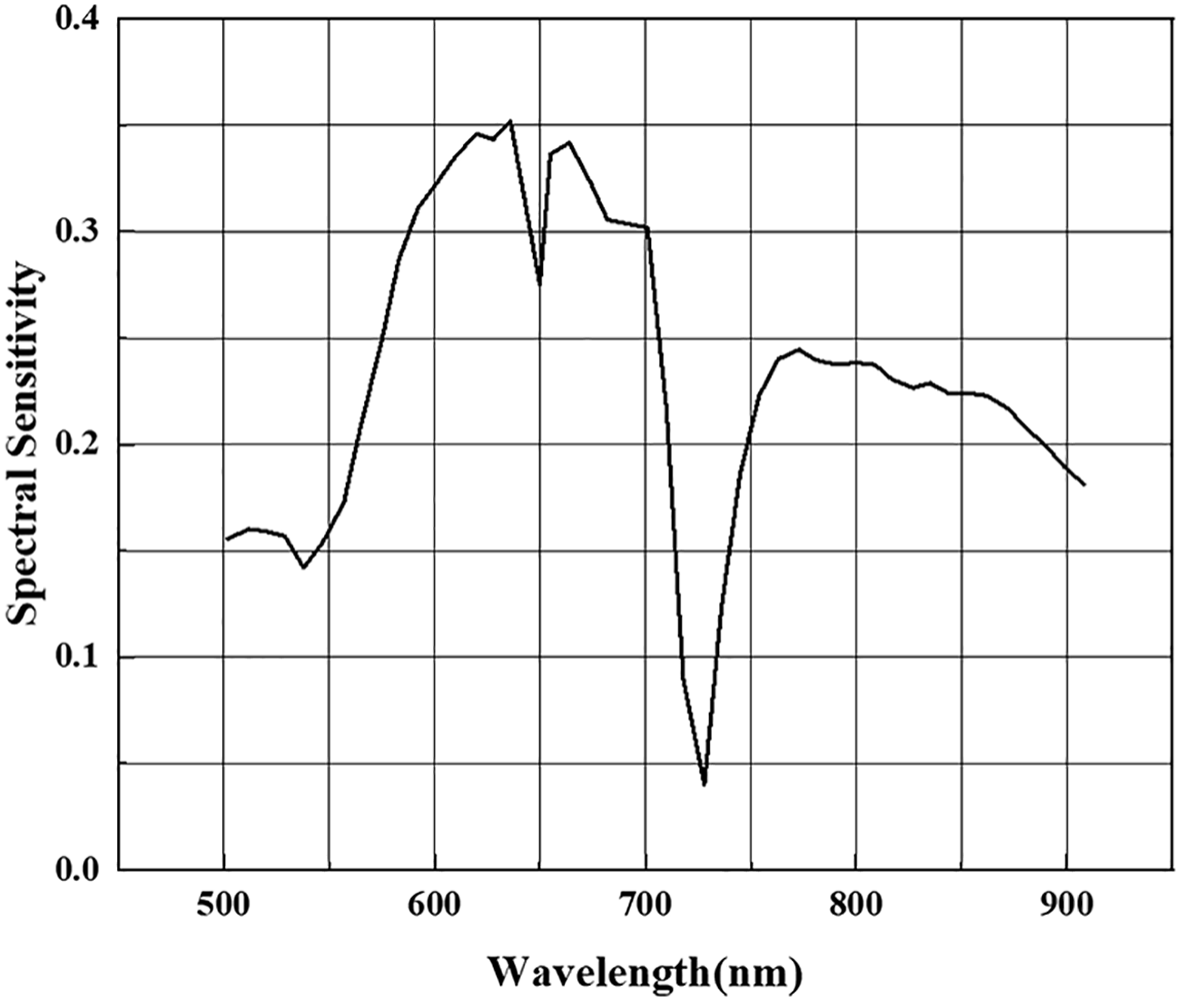
Spectral sensitivity of jujube spider mite infestation in each waveband.

In the feature selection process, we extracted different combinations of spectral bands using spectral sensitivity with ASP. **Figure 7** shows that the spectral sensitivity of each band was calculated separately and the absolute value was taken. The feature bands were selected with the two constraints of a large spectral sensitivity and a long inter-band distance interval. To ensure the effectiveness of the clustering algorithm, the dimension of the constructed jujube spider mite feature spectral space cannot be too high—that is, there cannot be too many bands in the combination of feature bands.

**Figure 8:**
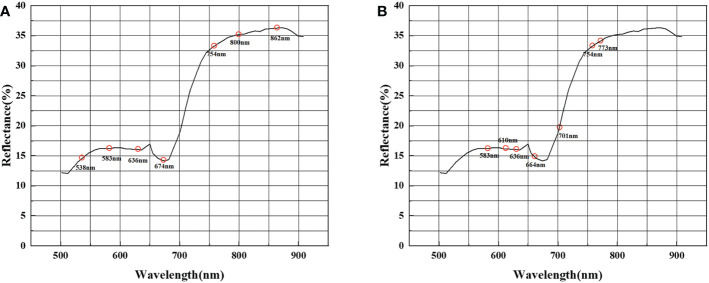
Combination of feature bands selected. **(A)** Combination of feature bands selected by spectral sensitivity, **(B)** combination of feature bands selected by band clustering.

We selected a total of seven bands of spectral reflectance at 538, 583, 636, 674, 754, 800 and 862 nm as the bands sensitive to jujube trees infected with spider mites based on the constraints of high spectral sensitivity and long band intervals (**Figure 8A**). In the band clustering process, the bands were clustered according to the amount of band information using the ASP method. We also calculated the reflectance band combinations for a total of seven bands at 583, 610, 636, 664, 701, 754 and 773 nm (**Figure 8B**).

### 3.3 Establishment and evaluation of the model for the recognition of jujube spider mite pests

We evaluated the recognition accuracy of the jujube pest recognition models constructed by different spectral feature construction methods combined with different clustering algorithms. Four different spectral features were extracted using PCA, LLE, spectral sensitivity and ASP. We then used three clustering methods (K-means, FCMs and density peak clustering) to establish PCA–K-means, PCA–FCM, PCA–DP, LLE–K-means, LLE–FCM, LLE–DP, SS–K-means, SS–FCM, SS–DP, BC–K-means, BC–FCM and BC–DP for jujube spider mite pest recognition and evaluated their recognition effects.

**Figure 9:**
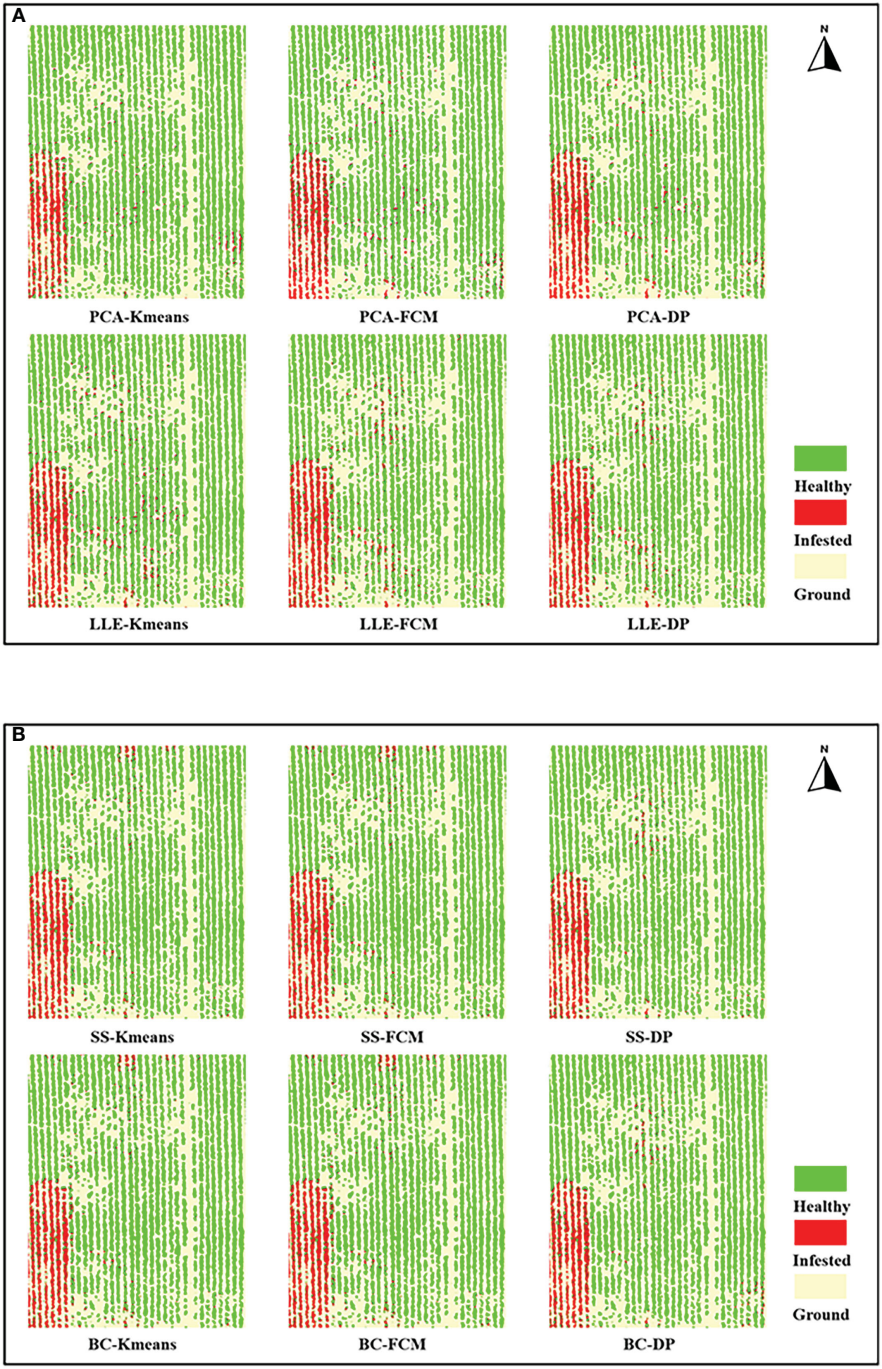
Recognition results mapping of jujube spider mite infestations. **(A)** Recognition results mapping of feature extraction models, **(B)** Recognition results mapping of feature selection models.

**Figures 9A, B** show the results of the feature extraction and feature selection of the jujube tree pest recognition model mapping. The model recognition effect is generally consistent with the ground field survey: the southwestern area is unmanaged abandoned land with a serious outbreak of jujube tree pests, whereas the rest of the area contains jujube trees that are generally healthy, with only sporadic infestations of spider mites.

**Figure 10:**
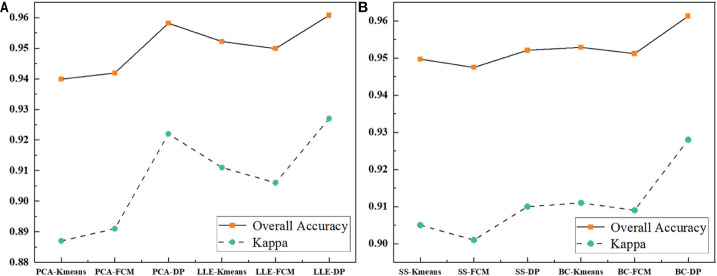
Comparison of the evaluation results of each model. **(A)** Comparison of the evaluation results of feature extraction models, **(B)** Comparison of the evaluation results of feature selection models.

**Table 2** record the results of the different recognition models of jujube spider mite pests. In general, the recognition methods of constructing the spectral–spatial features of jujube trees and clustering were highly accurate, with overall accuracies >93% and kappa coefficients >0.8. The BC–DP and LLE–DP recognition models were the best for the recognition of infected jujube trees, with an overall accuracy >96% and kappa coefficients >0.92. The overall accuracy and kappa coefficient for spider mite pest recognition were higher than those of the other models. By contrast, the PCA–K-means and PCA–FCM recognition models had the lowest accuracy for jujube spider mite pest recognition, with an overall classification accuracy of 94% and kappa coefficients of about 0.89. Among the different spectral feature selection methods, the band clustering and nonlinear dimensionality reduction algorithms achieved better results than the relatively low overall recognition accuracy of spectral sensitivity and PCA (**Figures 10A, B**). Among the different clustering methods, the density peak clustering algorithm was significantly more effective than the K-means and fuzzy C-means algorithms, whereas the K-means and FCMs algorithms were only approximately accurate in the recognition of jujube spider mite pests.

**Table 2:** Accuracy evaluation of each model on the recognition results of infected jujube trees.

DR	Cluster		Healthy	Infected	Ground	Sum	U(%)	OA(%)	Kappa
**PCA**	**K-means**	**Healthy**	4972	465	0	5437	91.4	**93.99**	**0.887**
		**Infected**	0	1378	0	1378	100		
		**Ground**	64	595	11213	11872	94.4		
		**Sum**	5036	2438	11213	18687			
		**P(%)**	98.7	56.5	100				
	**FCM**	**Healthy**	4972	425	0	5397	92.1	**94.19**	**0.891**
		**Infected**	0	1417	0	1417	100		
		**Ground**	64	596	11213	11873	94.4		
		**Sum**	5036	2438	11213	18687			
		**P(%)**	98.7	58.1	100				
	**DP**	**Healthy**	4841	0	0	4841	100	**95.82**	**0.922**
		**Infected**	6	1852	0	1858	99.7		
		**Ground**	189	586	11213	11988	93.5		
		**Sum**	5036	2438	11213	18687			
		**P(%)**	96.1	76	100				
**LLE**	**K-means**	**Healthy**	4703	66	0	4769	98.6	**95.22**	**0.911**
		**Infected**	113	1878	0	1991	94.3		
		**Ground**	220	494	11213	11927	94		
		**Sum**	5036	2438	11213	18687			
		**P(%)**	93.3	77	100				
	**FCM**	**Healthy**	4647	62	0	4709	98.7	**94.99**	**0.906**
		**Infected**	149	1891	0	2040	92.7		
		**Ground**	240	485	11213	11938	93.9		
		**Sum**	5036	2438	11213	18687			
		**P(%)**	92.3	77.6	100				
	**DP**	**Healthy**	4757	2	0	4759	99.9	**96.08**	**0.927**
		**Infected**	111	1984	0	2095	94.7		
		**Ground**	168	452	11213	11833	94.7		
		**Sum**	5036	2438	11213	18687			
		**P(%)**	94.4	81.3	100				
**SS**	**K-means**	**Healthy**	4852	228	0	5080	95.5	**94.97**	**0.905**
		**Infected**	14	1682	0	1696	99.2		
		**Ground**	170	528	11213	11911	94.1		
		**Sum**	5036	2438	11213	18687			
		**P(%)**	96.3	69	100				
	**FCM**	**Healthy**	4775	0	0	4775	100	**94.75**	**0.901**
		**Infected**	14	1718	0	1732	99.2		
		**Ground**	247	720	11213	12180	91.3		
		**Sum**	5036	2438	11213	18687			
		**P(%)**	94.8	70.5	100				
	**DP**	**Healthy**	4635	0	6	4641	99.9	**95.21**	**0.91**
		**Infected**	153	1944	0	2097	92.7		
		**Ground**	248	488	11213	11949	93.8		
		**Sum**	5036	2432	11219	18687			
		**P(%)**	92	79.9	99.9				
**BC**	**K-means**	**Healthy**	4829	0	0	4829	100	**95.29**	**0.911**
		**Infected**	0	1764	0	1764	100		
		**Ground**	207	674	11213	12094	92.7		
		**Sum**	5036	2438	11213	18687			
		**P(%)**	95.9	72.4	100				
	**FCM**	**Healthy**	4666	56	0	4722	98.8	**95.12**	**0.909**
		**Infected**	142	1896	0	2038	93		
		**Ground**	228	486	11213	11927	94		
		**Sum**	5036	2438	11213	18687			
		**P(%)**	92.7	77.8	100				
	**DP**	**Healthy**	4908	106	0	5014	97.8	**96.13**	**0.928**
		**Infected**	0	1843	0	1843	100		
		**Ground**	128	489	11213	11830	94.8		
		**Sum**	5036	2438	11213	18687			
		**P(%)**	97.5	75.6	100				

The bold values provided are the main reference standard for accuracy evaluation (OA and Kappa).

### 3.4 Analysis of recognition accuracy of different spectral feature selection and extraction algorithms

**Figure 11:**
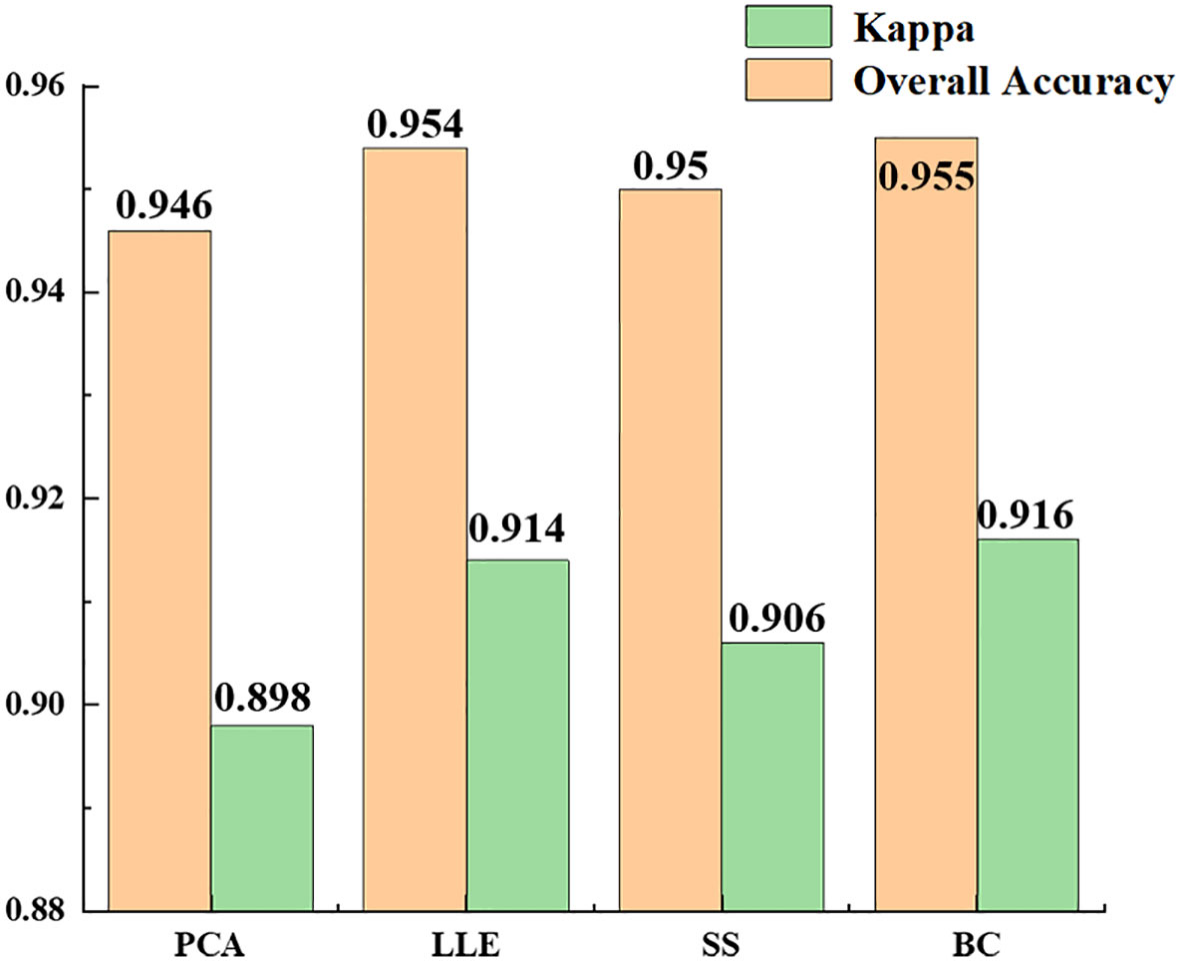
Comparison of recognition results of different spectral features.

**Table 3** shows the experimental results of the statistically different spectral feature construction methods, in which the overall accuracy of the recognition results obtained using PCA, LLE, spectral sensitivity, band clustering and other dimensionality reduction methods were 94.6, 95.4, 95 and 95.5%, respectively, and the kappa coefficients were 0.898, 0.914, 0.906 and 0.916, respectively. The results (**Figure 11**) showed that band clustering achieved the best results in the hyperspectral jujube spider mite pest recognition model, with the highest overall recognition accuracy and kappa coefficient. The recognition accuracy of the nonlinear dimensionality reduction method LLE was slightly lower than that of band clustering, which was also a better feature construction method, but the computational complexity of its solution was generally higher than that of band clustering due to the complexity and nonlinearity of the hyperspectral data. Spectral sensitivity is a classic and extensively experimentally validated spectral band selection method for pests and diseases, with an accuracy of 95% for jujube spider mite pest recognition, which was lower than the band clustering and nonlinear dimensionality reduction LLE methods. The lowest recognition accuracy was achieved by PCA, one of the most commonly used hyperspectral feature extraction methods, with an overall accuracy of about 94.6% for the recognition of jujube spider mite pests. Overall, the use of feature selection and extraction algorithms to construct spectral features to recognize jujube spider mite pests was very effective and the recognition accuracy was >94% in all cases, with the highest overall accuracy and kappa coefficient for waveband clustering in cross-sectional comparison with other methods. This was therefore the optimum method for constructing spectral features.

**Table 3:** Accuracy evaluation of recognition results of different spectral feature extraction methods (OA and Kappa of Table 3 is the accuracy evaluation of all recognition algorithms using some kind of dimensionality reduction).

DR		Healthy	Infected	Ground	Sum	U(%)	OA(%)	Kappa
**PCA**	**Healthy**	14785	890	0	15675	94.3	**94.6**	**0.898**
	**Infected**	6	4647	0	4653	99.8		
	**Ground**	317	1777	33639	35733	94.1		
	**Sum**	15108	7314	33639	56061			
	**P(%)**	97.8	63.5	100				
**LLE**	**Healthy**	14107	130	0	14237	99.1	**95.4**	**0.914**
	**Infected**	373	5753	0	6126	93.9		
	**Ground**	628	1431	33639	35698	94.2		
	**Sum**	15108	7314	33639	56061			
	**P(%)**	93.3	78.7	100				
**SS**	**Healthy**	14262	228	6	14496	98.4	**95**	**0.906**
	**Infected**	181	5344	0	5525	96.7		
	**Ground**	665	1736	33639	36040	93.3		
	**Sum**	15108	7308	33645	56061			
	**P(%)**	94.4	73.1	99.9				
**BC**	**Healthy**	14403	162	0	14565	98.8	**95.5**	**0.916**
	**Infected**	142	5503	0	5645	97.5		
	**Ground**	563	1649	33639	35851	93.8		
	**Sum**	15108	7314	33639	56061			
	**P(%)**	95.3	75.2	100				

The bold values are the main reference standard for accuracy evaluation (OA and Kappa).

### 3.5 Analysis of recognition accuracy of different clustering algorithms

**Figure 12:**
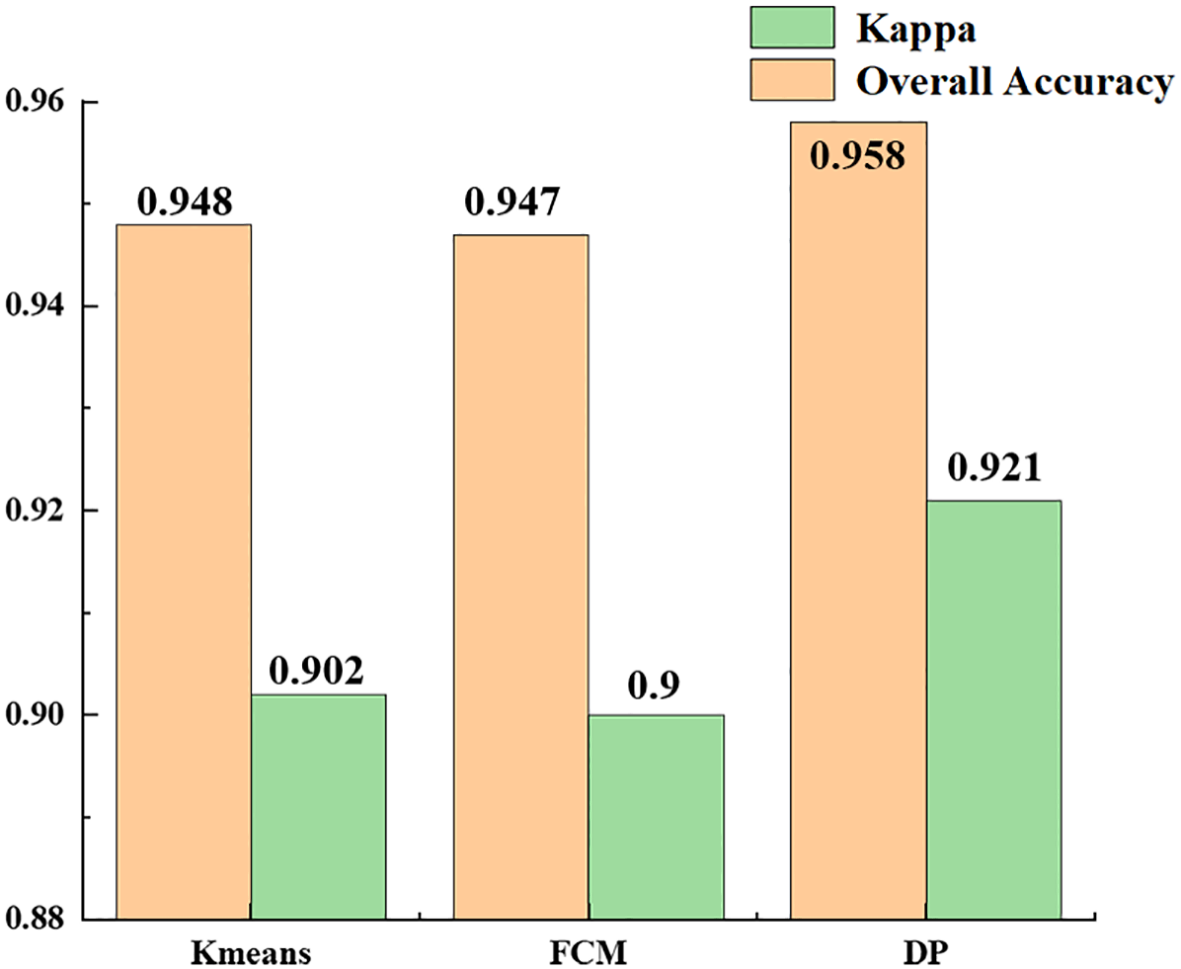
Comparison of recognition results of different clustering algorithms.

**Table 4** shows the statistical results of all the experiments using the same clustering algorithm. The recognition accuracy of the results obtained using the K-means, FCM and density peak clustering algorithms were 94.8, 94.7 and 95.8%. The results (**Figure 12**) show that density peak clustering was the optimum clustering method in the hyperspectral infected jujube tree recognition model, with a kappa coefficient of 0.921 for the recognition results, which is 2% better than the K-means-FCM method. K-means is a classic clustering algorithm widely used in the classification of remote sensing images; fuzzy clustering has been increasingly used in remote sensing and other research fields in recent years with the development of fuzzy mathematics. The difference between K-means and fuzzy clustering in the accuracy of jujube leaf mite pest recognition was not significant and the kappa coefficients were both about 0.9. The K-means and FCM algorithms are distance-based clustering methods. When dealing with a large amount of data, the clustering results of K-means and FCM are susceptible to the influence of the initial values, which restricts them from achieving a high clustering accuracy. The density peak algorithm first calculates the density of data points to obtain the centers of clusters, which improves the overall efficiency and performance of the clustering process.

**Table 4:** Accuracy evaluation of the recognition results of different clustering methods.

Clustering		Healthy	Infected	Ground	Sum	U(%)	OA(%)	Kappa
**K-means**	**Healthy**	19356	759	0	20115	96.2	**94.8**	**0.902**
	**Infected**	127	6702	0	6829	98.1		
	**Ground**	661	2291	44852	47804	93.8		
	**Sum**	20144	9752	44852	74748			
	**P(%)**	96.1	68.7	100				
**FCM**	**Healthy**	19060	543	0	19603	97.2	**94.7**	**0.9**
	**Infected**	305	6922	0	7227	95.8		
	**Ground**	779	2287	44852	47918	93.6		
	**Sum**	20144	9752	44852	74748			
	**P(%)**	94.6	71	100				
**DP**	**Healthy**	19141	108	0	19249	99.4	**95.8**	**0.921**
	**Infected**	270	7623	0	7893	96.6		
	**Ground**	733	2015	44852	47600	94.2		
	**Sum**	20144	9746	44852	74742			
	**P(%)**	95	78.2	100				

The bold values are the main reference standard for accuracy evaluation (OA and Kappa).

## 4 Discussion

### 4.1 Feasibility analysis of hyperspectral images for recognizing spider mite pests in jujube trees

In pest and disease monitoring, a high spatial resolution makes it possible to obtain agronomic parameters at fine scales, whereas a high spectral resolution can capture subtle spectral changes caused by crop stress as a result of pests and diseases. We extracted the spectral profiles of healthy jujube trees and trees infested by jujube spider mite. The spectral profiles of jujube trees infested with spider mites had a significantly higher reflectance in the visible range and a lower reflectance in the near-infrared range than healthy jujube trees. This is due to the damage to the chlorophyll and canopy cell tissues inside the jujube tree as a result of stress from the spider mite infestations.

In traditional pest monitoring, the spectral features are mainly characterized by the vegetation index of crops, which is a combination of the reflectance of different features at multiple wavelengths. In multispectral remote sensing, the vegetation index is a robust and effective way of extracting spectral features limited by the number of wavelengths, but it cannot fully exploit the spectral information in hyperspectral images from the full wavelength range. Although some researchers have extracted the optimum spectral bands of plants using algorithms such as the continuous projection transform, there is still a lack of evaluation of the optimum methods for constructing spectral features.

We evaluated four methods of constructing spectral features—PCA, LLE, spectral sensitivity and band clustering—with final recognition accuracies of 94.6, 95.4, 95 and 95.5%, respectively. All the dimensionality reduction methods, apart from PCA, achieved an accuracy of >95% for recognition. The accuracy of the nonlinear dimensionality reduction method LLE was significantly higher than that of PCA, which may be caused by the composition of the hyperspectral data, the high-dimensional characteristics of the hyperspectral data, information redundancy and the physical properties of object scattering. Dimensionality reduction of hyperspectral data using linear models usually results in some errors because they are usually nonlinear, resulting in a hyperspectral structure that also exhibits nonlinearity (Shuangping et al., 2015). The accuracy of feature selection is slightly higher than that of feature extraction because feature extraction is based on the amount of information between bands. Although the spectra of healthy and stressed jujube trees are different, the spectral curves are similar. Although feature extraction is commonly achieved based on the difference in information between bands—in addition to the influence of weeds, shadows and other factors—the effect of the spectral features extracted under the approximate spectral features will be reduced after clustering, which also leads to. In this experiment, the overall recognition accuracy of spectral feature extraction was lower than that of feature selection. Feature selection may achieve better results when the spectral differences of the recognized features are large.

Extracting spectral–spatial features that effectively distinguish healthy jujube trees from those infested with spider mites is a key issue recognizing jujube spider mite infestations. Previous studies either focused on only the spectral features of vegetation and neglected the spatial information in images, or the texture features were only introduced as a supplement with a gray level co-occurrence matrix (Guo et al., 2020). We used a weighted spatial–spectral mean filtering method to incorporate spatial information from the hyperspectral data into the spectral features, providing a new way to effectively use spectral–spatial information in pest and disease monitoring.

### 4.2 Analysis of the main factors affecting the accuracy of jujube spider mite recognition

Spectral reflectance is the most important factor affecting the recognition of jujube tree pests. The reflectance of healthy and infected jujube trees will change as a result of the influence of environmental factors such as light and temperature and the hyperspectral sensor will show deviations in the radiation information recorded from jujube trees.

Unlike low-growing plants (e.g., wheat and rice), when sensors record radiation information from forest trees such as jujube trees, they are inevitably affected by shadows caused by shading and the spectral reflectance is between the ground and healthy jujube trees, which can cause a large number of misclassifications. These factors cause a large amount of pepper noise, which affects the recognition accuracy of the model. To reduce the errors caused by shadows and mismatches between the object and its spectra, we used weighted spatial–spectral mean filtering to combine the recognized objects with the spectral reflectance of their neighbors. This makes full use of the spatial correlation between the pixel points and reduces the influence of a large amount of pepper noise on the recognition results.

The background also reduces the recognition accuracy. When the UAV acquires ground images, objects such as bare ground, weeds, agricultural facilities and their cluttered spectral characteristics can cause bias in the recognition results. When clusters of clusters increase, this also increases the time and result complexity of the clustering algorithms. We compared the reflection characteristics of different objects and used the spectral index to extract the surface background uniformly, which greatly reduced the influence of the surface background on the recognition accuracy.

Another influencing factor is the error caused by the high-dimensional characteristics of hyperspectral data itself, which has a large amount of redundant information and strong correlations between adjacent bands. The constraints of high dimensionality in machine learning and deep learning based on distance metrics can reduce the classification accuracy when dealing with high-dimensional data. We adopted the method of dimensionality reduction to extract spectral features after data pre-processing, which reduced the data volume and data dimensionality. We compared the effects of feature selection and feature extraction and four methods (the linear dimensionality reduction algorithm, the nonlinear dimensionality reduction algorithm, spectral sensitivity and band clustering) on the recognition of jujube spider mite pests. We verified that using spectral–spatial features combined with clustering can achieve a good accuracy in jujube spider mite pest recognition.

Although the acquired hyperspectral images have both a high spectral resolution and high spatial resolution, there are still a large number of mixed pixels. The spectral reflectance of mixed pixels is usually between the spectral reflectance of the two types of objects, which can cause a large number of misclassifications. The more mixed pixels there are, the larger the error, especially when the spatial resolution is lower. Although the spatial information has weakened the influence of mixed pixels, they are still one of the main factors limiting the recognition accuracy.

### 4.3 Deficiencies and improvement directions

We explored the high-precision recognition of jujube spider mite infestations on a regional scale using UAV hyperspectral data. Low-altitude remote sensing is highly accurate, but it is also limited by time, space, funding and other factors, and there is still a need to develop three-dimensional, accurate, real-time, wide-scale and all-round pest monitoring systems.

The traditional clustering algorithms still have the defects of low computational efficiency and poor noise immunity. Similarity-constrained subspace clustering algorithm achieves fast clustering of hyperspectral images by sparse matrix and spatial filtering, which greatly reduces the clustering time of hyperspectral images and effectively improves the clustering performance (Hinojosa et al., 2021). Subspace clustering *via* dictionary learning with adaptive regularization uses dictionary modeling to achieve effective subspace clustering, which improves the robustness of HIS against noise and spectral variation (Huang et al., 2021). Algorithms mentioned above have good promise in improving the computational efficiency and noise resistance of clustering algorithms for recognizing spider mite infestations, these advanced clustering algorithms could be applied in subsequent studies.

For data processing, we used the clustering method in unsupervised learning due to the high dimensionality of hyperspectral data and the difficulty of obtaining ground sample points. After increasing the number of ground samples and further improving the data quality, supervised learning and deep learning methods were used to construct the recognition model.

In terms of data source resolution, the UAV-mounted spectrometer has a high accuracy, but is limited by regional and cost factors. Achieving large-scale, real-time pest monitoring requires a satellite-scale pest recognition model and the establishment of a link between aerial and satellite measurements.

At present, there are few available hyperspectral satellites and their spatial resolution is low. It is worth exploring whether using a multispectral satellite with a low spectral and high spatial resolution for pest recognition and monitoring will achieve better results. We need to find the optimum balance of spectral and spatial resolution to achieve the optimum solution for pest recognition modeling.

The mixed image element problem is an important factor limiting the classification accuracy of remote sensing images. At present, there has been much research on mixed image decomposition. An important direction to improving the accuracy of pest recognition needs to consider how to integrate the mixed image decomposition algorithm into the disease recognition model or to reduce the error caused by the mixed image to an acceptable range.

## 5 Conclusions

We have presented a spider mite pest recognition model based on constructing the spectral features of jujube trees through feature extraction and feature selection, incorporating spatial information into the images using weighted spatial–spectral mean filtering and recognizing spider mite infestations of jujube trees using clustering. Through experimental analysis, we showed that this recognition model has a high recognition accuracy (>93%) for jujube spider mite infestations. The BC–DP algorithm was the best model for jujube mite pest recognition, with an overall accuracy of 96.13% and a kappa coefficient of 0.928. This recognition model is not limited to the recognition of jujube spider mite infestations and could be extended to the recognition of other crop diseases and object classification, although further experimental verification is required.

## Data availability statement

The datasets presented in this article are not readily available because Use permissions of this dataset belongs to Xinjiang Production and Construction Corps. Requests to access the datasets should be directed to 2020110667@sdau.edu.cn.

## Author contributions

YW conceptualized the experiment, selected the algorithms, collected and analyzed the data, and wrote the manuscript. HQ trained the algorithms, XZ and PW collected and analyzed data, and wrote the manuscript. QZ and XL supervised the project. All authors contributed to the article and approved the submitted version.
